# Tumour cells down-regulate CCN2 gene expression in co-cultured fibroblasts in a Smad7- and ERK-dependent manner

**DOI:** 10.1186/1478-811X-11-75

**Published:** 2013-10-03

**Authors:** Beverley A van Rooyen, Georgia Schäfer, Virna D Leaner, M Iqbal Parker

**Affiliations:** 1 International Centre for Genetic Engineering and Biotechnology (ICGEB), Anzio Rd Observatory, Cape Town 7925, South Africa; 2 Division of Medical Biochemistry & the Institute of Infectious Diseases and Molecular Medicine, Faculty of Health Sciences, University of Cape Town, Cape Town, South Africa; 3 Present address: Division of Haematology, Faculty of Medicine and Health Sciences, University of Stellenbosch, Franzie van Zyl Drive, Tygerberg 7505, South Africa

**Keywords:** Feedback regulation, CCN2, Type 1 collagen, Signal transduction, Host-tumour cell interaction, Breast cancer

## Abstract

**Background:**

Recent studies have revealed that interactions between tumour cells and the surrounding stroma play an important role in facilitating tumour growth and invasion. Stromal fibroblasts produce most of the extracellular matrix components found in the stroma. The aim of this study was to investigate mechanisms involved in tumour cell-mediated regulation of extracellular matrix and adhesion molecules in co-cultured fibroblasts. To this end, microarray analysis was performed on CCD-1068SK human fibroblast cells after direct co-culture with MDA-MB-231 human breast tumour cells.

**Results:**

We found that the expression of both connective tissue growth factor (CTGF/CCN2) and type I collagen was negatively regulated in CCD-1068SK fibroblast cells under direct co-culture conditions. Further analysis revealed that Smad7, a known negative regulator of the Smad signalling pathway involved in CCN2 promoter regulation, was increased in directly co-cultured fibroblasts. Inhibition of Smad7 expression in CCD-1068SK fibroblasts resulted in increased CCN2 expression, while Smad7 overexpression had the opposite effect. Silencing CCN2 gene expression in fibroblasts led, in turn, to a decrease in type I collagen mRNA and protein levels. ERK signalling was also shown to be impaired in CCD-1068SK fibroblasts after direct co-culture with MDA-MB-231 tumour cells, with Smad7 overexpression in fibroblasts leading to a similar decrease in ERK activity. These effects were not, however, seen in fibroblasts that were indirectly co-cultured with tumour cells.

**Conclusion:**

We therefore conclude that breast cancer cells require close contact with fibroblasts in order to upregulate Smad7 which, in turn, leads to decreased ERK signalling resulting in diminished expression of the stromal proteins CCN2 and type I collagen.

## Background

In normal mammary tissue, epithelial cells form ducts and glands that are separated from the surrounding connective tissue by a basement membrane. The connective tissue, or stroma, is made up of fibrillar extracellular matrix (ECM), capillaries and cells such as fibroblasts, immune and inflammatory cells [1,2] and serves as a barrier that impedes tumour development [3-5]. However, complex tumour-stromal interactions may result in changes to the stroma that facilitate breakdown of the basement membrane and allows tumour cells to invade the surrounding ECM. Here, the tumour cells interact with both ECM components and stromal cells in a way that would not occur under normal conditions, and this may facilitate further tumour invasion and metastasis [3,5-10]. Stromal fibroblasts are responsible for synthesizing and depositing most of the ECM components and, therefore, interactions between tumour cells and fibroblasts play an important role in determining how tumour cells alter the ECM to facilitate tumour invasion.

Structural proteins such as collagen, fibronectin and laminin make up a large proportion of the ECM. However, another group of proteins known as matricellular proteins are also found associated with the ECM. Matricellular proteins do not play a direct role in maintaining physical structure but are rather involved in modulating and co-mediating cellular responses through interactions with cell surface receptors, growth factors, cytokines and matrix proteins [11,12]. Connective tissue growth factor (CTGF) or CCN2 is a member of the CCN family of matricellular proteins and mainly acts through interactions with cell adhesion receptors such as integrins and heparin sulfate proteoglycans (HSPGs) [12]. CCN2 expression is regulated mainly at the transcriptional level [13,14] and one of the most potent inducers of CCN2 gene expression in fibroblasts, but not in epithelial cells, is transforming growth factor beta (TGFβ) [14,15]. Regulation of CCN2 gene expression by TGFβ involves the association of a Smad3/Smad4 complex with a Smad binding element (SBE) on the CCN2 promoter [16]. The CCN2 promoter also has a TGFβ response element (TGFβRE) which appears to be important for the regulation of basal CCN2 gene expression in fibroblasts, and is therefore also called the basal control element (BCE-1) [16]. Other signalling pathways that are involved in basal and TGFβ-mediated CCN2 up-regulation include the Ras/MEK/ERK and protein kinase C (PKC) pathways [14,17].

CCN2 is thought to act mainly as a co-mediator of TGF-β’s ability to promote type I collagen synthesis, as ccn2-/- embryonic fibroblasts were unable to induce type I collagen synthesis in response to TGFβ [18]. An important relationship therefore exists between TGFβ, CCN2 and type I collagen, and in aged human skin (≥ 80 years) the expression of all three of these proteins is co-ordinately reduced when compared to levels in younger skin samples (21–30 years) [19].

Current knowledge of the role tumour cells play in regulating the expression of various components of the ECM in the tumour environment is limited. In this study we investigated this further by using microarray technology to measure changes in the expression of ECM components and adhesion molecules in human fibroblasts that were co-cultured with human breast tumour cells. We show that MDA-MB-231 breast tumour cells negatively regulate CCN2 and type I collagen gene expression in CCD-1068SK fibroblasts in a Smad7-dependent manner through decreased activation of the MEK/ERK signalling pathway. This effect was only observed in CCD-1068SK fibroblasts that were directly co-cultured with MDA-MB-231 tumour cells, suggesting that breast tumour cells require close contact with fibroblasts in the tumour microenvironment to influence the expression of ECM components.

## Results

### The effect of tumour cell/fibroblast co-culture on ECM and adhesion molecule gene expression

To investigate the effect of close contact with tumour cells on the expression of cell adhesion and ECM components in fibroblasts, cells were directly co-cultured and subsequently separated before further gene expression analysis. CCD-1068SK human fibroblasts pre-labelled with PKH-67 green fluorescent dye were mixed with an equal number of MDA-MB-231 human breast tumour cells, co-cultured for 48 hours and separated from the tumour cells by FACS for subsequent RNA isolation to profile the expression of several ECM genes by means of the Oligo GEArray® Human Extracellular Matrix and Adhesion Molecules microarray (SABiosciences) (Additional file 1: Table S1). The array analysis showed that direct co-culture with MDA-MB-231 tumour cells led to an increase in the expression of matrix metalloprotease 1 (MMP1) in CCD-1068SK fibroblasts relative to CCD-1068SK mono-cultures while the expression of a number of collagen genes was down-regulated (Table 1). Interestingly, the expression of connective tissue growth factor (CTGF/CCN2) was substantially decreased in co-cultured fibroblasts.

**Table 1:** Differential expression of genes in CCD-1068SK fibroblasts after co-culture with MDA-MB-231 breast tumour cells

**Gene name**	**Relative expression in co-cultures vs. mono-cultures [CCD(MDA)/CCD(CCD)]**
MMP1	1.26
COL1A1	0.50
COL18A1	0.40
COL6A3	0.36
COL12A1	0.29
COL4A2	0.18
COL8A1	0.14
COL5A3	0.05
CCN2	0.0001

**Figure 1:**
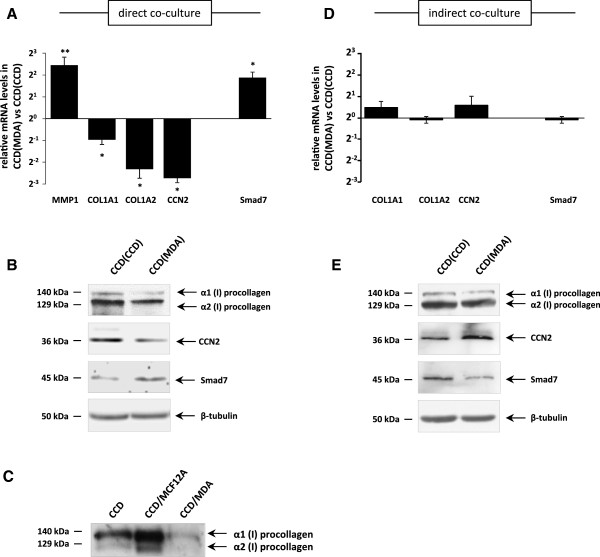
**MDA-MB-231 tumour cells influence the regulation of MMP1, type I collagen and Smad7 gene expression in CCD-1068SK fibroblasts during direct co-culture.** For direct co-culture experiments, CCD-1068SK fibroblasts were labelled with green fluorescent dye, PKH67, mixed with an equal number of MDA-MB-231 breast tumour cells and co-cultured for 48 hours in serum-free medium. Fibroblasts were separated from tumour cells by FACS and used for further analysis. CCD-1068SK monocultures were used as controls. **(A)** Quantitative real-time RT-PCR analysis of MMP1, COL1A1, COL1A2, CCN2 and Smad7 mRNA levels in co-cultured CCD-1068SK fibroblasts. The graph shows the mean ± SD from a representative experiment, relative to GAPDH (*p ≤ 0.05, n = 3). **(B)** Western blotting results show endogenous α1(I) and α2(I) procollagen, CCN2 and Smad7 protein levels in CCD-1068SK fibroblasts after direct co-cultures. β-tubulin was used as a loading control. **(C)** De novo synthesis of type I collagen measured by [3H]-proline incorporation into CCD-1068SK/MDA-MB-231 direct co-culture medium. CCD-1068SK fibroblasts monocultures and co-cultures with non-tumourigenic MCF12A cells were used as controls. The medium was run on an 8% SDS-PAGE gel and exposed to film for 7 days at -80°C, before being developed. **(D)** Quantitative real-time RT-PCR of indirectly co-cultured cells as described show relative COL1A1, COL1A2, CCN2 and Smad7 mRNA levels in CCD-1068SK fibroblasts. The graph shows the mean ± SD from a representative experiment (n = 3). **(E)** Western blotting results show endogenous α1(I) and α2(I) procollagen levels, CCN2 and Smad7 in CCD-1068SK fibroblasts after indirect co-culture with tumour cells. β-tubulin was used as a loading control. Abbreviations: CCD, CCD-1068SK; MDA, MDA-MB-231. Brackets enclose the cell line with which CCD-1068SK fibroblasts were co-cultured.

The microarray findings for MMP1, COL1A1, COL1A2 and CCN2 were independently confirmed by quantitative real-time RT-PCR, showing that MMP1 gene expression was significantly up-regulated while COL1A1, COL1A2 and CCN2 mRNA levels were significantly decreased in fibroblasts that were co-cultured with tumour cells (Figure 1A). Both CCN2 and type I collagen are known to be positively regulated in response to TGFβ via the Smad signalling pathway [14,20] and, since both CCN2 and type I collagen were negatively regulated in fibroblasts in response to tumour cell co-culture, we investigated the expression of the negative regulator of TGFβ signalling, Smad7. Indeed, Smad7 gene expression was significantly increased in co-cultured compared to mono-cultured fibroblasts (Figure 1A). These findings were further supported by Western Blot analysis showing that Smad7 protein was elevated in co-cultured fibroblasts while both CCN2 and type I collagen levels were decreased (Figure 1B). The secretion of radioactively labeled α1(I) and α2(I) procollagen chains synthesized by CCD-1068SK fibroblasts during co-culture with MDA-MB-231 cells was investigated by adding [^3^H]-proline to the culture medium during the period of co-culture. We found lower levels of exogenous α1(I) and α2(I) procollagen in the medium from CCD-1068SK/MDA-MB-231 co-cultures compared to levels in CCD-1068SK monocultures or CCD-1068SK co-cultured with MCF12A breast epithelial cells that served as a benign control (Figure 1C). These results suggest that, when in direct contact with fibroblasts, MDA-MB-231 tumour cells were able to negatively regulate the expression of certain ECM components in CCD-1068SK fibroblasts, including CCN2 and type I collagen. This regulation may occur through up-regulation of the negative regulator, Smad7.

Tumour cells may be communicating with fibroblasts in a paracrine manner by secreting soluble factors such as cytokines and growth factors that can modulate Smad7, CCN2 and type I collagen gene expression in neighbouring fibroblasts via such secreted factors. To investigate this possibility, an indirect co-culture system was used in which CCD-1068SK fibroblasts were separated from the MDA-MB-231 tumour cells using a transwell insert with a 0.2 μm pore size. This allowed secreted factors to pass through but prevented direct contact between fibroblasts and tumour cells. Analysis of gene expression by quantitative real-time RT-PCR in indirectly co-cultured CCD-1068SK fibroblasts revealed that tumour cells did not influence the expression of COL1A1, COL1A2, CCN2 or Smad7 when compared to fibroblast monocultures (Figure 1D). In fact, Western Blot analysis revealed that CCN2 protein levels were increased while Smad7 was decreased (Figure 1E). These results suggest that tumour cell-mediated regulation of Smad7, CCN2 and type I collagen expression in fibroblasts was dependent on the contacts with or close proximity of the tumour cells to these fibroblasts.

### Smad7 influences the expression of CCN2 and type I collagen gene expression

**Figure 2:**
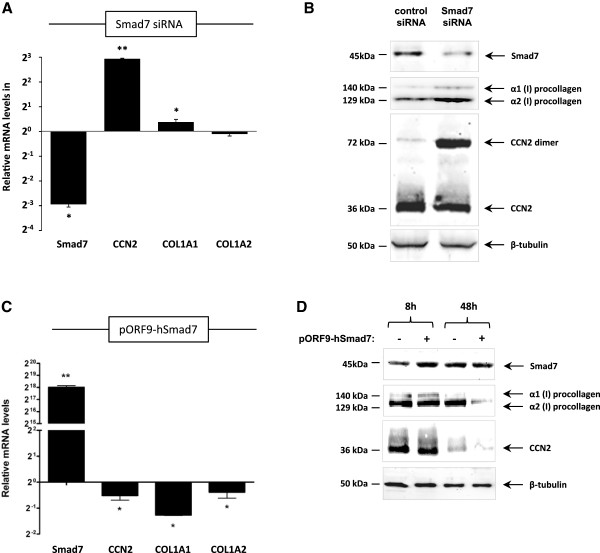
**Differential Smad7 expression leads to changes in the expression of CCN2 and type I collagen in CCD-1068SK fibroblasts.** CCD-1068SK fibroblasts were transfected with 80 μM Smad7 siRNA and incubated for 48 hours before isolating RNA and protein for further analysis. Fibroblasts transfected with scrambled siRNA were used as a control. **(A)** Quantitative real-time RT-PCR results show levels of Smad7, CCN2, COL1A1 and COL1A2 mRNA relative to GAPDH (*p ≤ 0.05, ** p ≤ 0.01, n = 3). **(B)** Western blotting results show Smad7, α1(I) and α2(I) procollagen and CCN2 levels in Smad7 knock-down fibroblasts compared to control fibroblasts. The CCN2 antibody detected a band at 36-38 kDa and 72 kDa that represent monomeric and dimeric forms of the protein. **(B)** For Smad7 overexpression experiments, CCD-1068SK fibroblasts were transiently transfected with 1 μg of the pORF9-hSmad7 plasmid. RNA and protein was isolated 8 hours and 48 hours after transfection. **(C)** Relative levels of Smad7, CCN2, COL1A1 and COL1A2 were quantified by means of quantitative real-time RT-PCR analysis (*p ≤ 0.05, ** p ≤ 0.01, n = 3). **(D)** Antibodies against Smad7, CCN2 and type I collagen were used to detect the respective protein levels by means of Western blot analysis. β-tubulin was used as a loading control.

To determine whether the observed increase in Smad7 was associated with decreased CCN2 and type I collagen levels, Smad7 gene expression in CCD-1068SK fibroblasts was altered by both gene silencing as well as transient overexpression. siRNA mediated knock-down of Smad7 in fibroblasts resulted in a substantial increase in both CCN2 mRNA and protein levels compared to controls (Figure 2A and B). Although all Western Blots were performed under denaturing conditions, we observed the appearance of both monomeric and dimeric forms of CCN2 protein at 36 kDa and 72 kDa, respectively, with a specific increase in CCN2 dimerization in Smad7 knock-down fibroblasts (Figure 2B). The levels of α1(I) and α2(I) procollagen were also increased in Smad7 knock-down fibroblasts compared to control fibroblasts, although only COL1A1 levels appeared to be affected at an mRNA level (Figure 2A and B).

Transfecting CCD-1068SK fibroblasts with the Smad7 overexpression plasmid pORF9-hSmad7 caused a significant decrease in CCN2, COL1A1 and COL1A2 mRNA levels (Figure 2C), which is in agreement with the expression data shown in Figure 1A. While Smad7 protein levels were found to peak 8 hours post transfection, the effect on CCN2 and type I collagen gene expression was only observed after 48 hours (Figure 2D). These results suggest that increased levels of Smad7 in CCD-1068SK fibroblasts can negatively affect the expression of both CCN2 and type I collagen, as observed in fibroblasts after direct co-culture with MDA-MB-231 tumour cells (Figure 1A and B).

### CCN2 is a positive regulator of type I collagen gene expression

**Figure 3:**
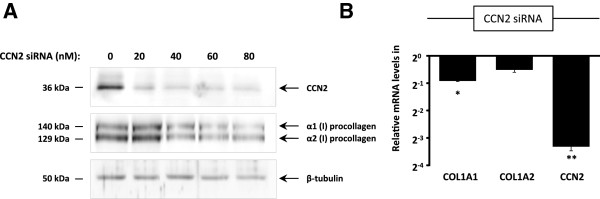
**CCN2 knock-down leads to decreased expression of type I collagen.** CCD-1068SK fibroblasts were transfected with 20 to 80 μM of CCN2 siRNA and incubated for 48 hours before isolating RNA and protein for further analysis. Fibroblasts transfected with scrambled siRNA were used as a control. **(A)** CCN2 and type I procollagen protein levels in CCN2 knock-down fibroblasts, analysed by Western blotting. β-tubulin was used as a loading control. **(B)** Real-time PCR results show relative COL1A1, COL1A2 and CCN2 mRNA levels, relative to GAPDH (*p ≤ 0.05, ** p ≤ 0.01, n = 3).

Previous studies have suggested that changes in CCN2 expression can affect type I collagen gene expression in fibroblasts [19,21]. We therefore investigated whether CCN2 knock-down in CCD-1068SK fibroblasts would have a downstream effect on type I collagen gene expression. CCD-1068SK fibroblasts were transfected with increasing concentrations of CCN2 siRNA and incubated for an additional 48 hours. Western blot analysis of the extracted protein showed that silencing CCN2 had a negative regulatory effect on both α1(I) and α2(I) procollagen gene expression (Figure 3A). CCD-1068SK fibroblasts transfected with 40 nM CCN2 siRNA were also subjected to quantitative real-time RT-PCR analysis, and showed an associated decrease in both COL1A1 and COL1A2 mRNA levels observed as a result of CCN2 knock-down (Figure 3B). Inhibition of CCN2 gene expression in CCD-1068SK fibroblasts therefore associates with decreased type I collagen expression in these cells.

### A role for ERK1/2 in the regulation of CCN2 and type I collagen gene expression

**Figure 4:**
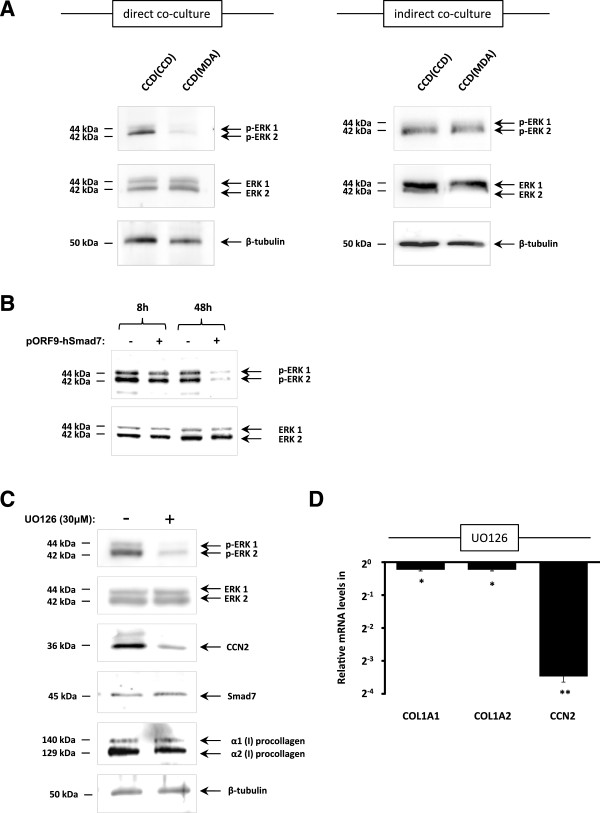
**Smad7-dependent decreased activation of ERK1/2 in CCD-1068SK fibroblasts directly co-cultured with MDA-MB-231. (A)** CCD-1068SK fibroblasts were directly or indirectly co-cultured with an equal number of MDA-MB-231 tumour cells and incubated for 48 hours. Levels of p-ERK 1/2 (T402, Y404) were measured in co-cultured CCD-1068SK fibroblasts by means of Western blot analysis and compared to levels in CCD-1068SK monocultures. ERK1/2 and β-tubulin levels were detected as experimental controls. **(B)** CCD-1068SK fibroblasts transfected with the pORF9-hSmad7 and overexpressing Smad7, as shown in Figure 2D, were used for further analysis of p-ERK 1/2 levels. ERK1/2 and β-tubulin served as controls. **(C)** The MEK/ERK inhibitor U0126 was added to CCD-1068SK fibroblasts and incubated for 48 hours. Protein was isolated for Western blot analysis of p-ERK 1/2 as well as CCN, Smad7 and type I procollagen. ERK1/2 and β-tubulin were used as controls. **(D)** Quantitative real-time RT-PCR was used to measure COL1A1, COL1A2 and CCN2 mRNA levels in CCD-1068SK fibroblasts incubated with U0126 inhibitor for 48 hours (*p ≤ 0.05, ** p ≤ 0.01, n = 3).

Previous studies have shown that the MEK/ERK signalling pathway is a positive regulator of CCN2 gene expression [14,17,22]. We therefore investigated whether changes in MEK/ERK signalling could account for the observed decreased CCN2 gene expression in CCD-1068SK fibroblasts co-cultured with MDA-MB-231 tumour cells. We found that direct, but not indirect, co-culture of fibroblasts with tumour cells led to a substantial decrease in phosphorylated ERK 1 and ERK 2 when compared to fibroblast monocultures (Figure 4A) while the levels of total ERK remained unchanged in both direct and indirect co-cultures. Since fibroblasts directly co-cultured with tumour cells were found to have elevated Smad7 gene expression with downstream effects on CCN2 and type I collagen (Figures 1 and 2), we therefore asked whether Smad7 affects activation of the ERK signalling pathway. We transiently transfected CCD-1068SK fibroblasts with pORF-hSmad7 and found that overexpression of Smad7 (as shown in Figure 2C) led to a decrease in activated ERK1 and ERK2, with very low levels of phosphorylated ERK1/2 observed 48 hours post transfection (Figure 4B).

To determine whether decreased activation of the MEK/ERK signalling pathway could be associated with decreased expression of CCN2 and type I collagen, CCD-1068SK fibroblasts were cultured in the presence of the MEK pathway inhibitor U0126. Western blot results showed that decreased ERK 1/2 phosphorylation resulted in a decrease in CCN2 protein and mRNA levels in CCD-1068SK fibroblasts (Figure 3C and D) while no significant effect was observed on COL1A1 and COL1A2 gene expression. These results suggest that the increase in Smad7 levels observed in directly co-cultured fibroblasts can negatively regulate MEK/ERK signalling which has downstream effects mainly on CCN2 expression.

## Discussion

It has recently been shown that genetic mutations are not the only factors that play a role in the progression of transformed epithelial cells to invasive tumour cells, but that continuous communication with the surrounding stroma may also facilitate tumour development [8,23,24]. If tumours progress to the invasive stage, the basement membrane which usually separates the tumour cells from the fibroblasts is degraded, allowing tumour cells to invade into the surrounding stroma where they come into close contact with stromal fibroblasts. Since these fibroblasts are the main producers of the components making up the ECM, close interactions with tumour cells could influence ECM production by these fibroblasts with further consequences for tumour migration and invasion.

In the present study we established an *in vitro* co-culture model of MDA-MB-231 breast tumour cells and normal CCD-1068SK breast skin fibroblasts and applied microarray analysis to identify the genes affected by direct cell-cell contact during culture. We showed that tumour cells are able to down-regulate the expression of ECM genes such as type I collagen and CCN2, while up-regulating the expression of collagenases such as MMP1 in neighbouring fibroblasts. Moreover, we identified Smad7 as a putative negative regulator of both CCN2 and type I collagen gene expression in fibroblasts, with Smad7 mRNA and protein levels being significantly increased in CCD-1068SK fibroblasts that were directly co-cultured with MDA-MB-231 tumour cells. Importantly, these effects were found to be a result of direct cell-cell contact and not mediated by growth factors or cytokines secreted into the medium, as shown by indirect co-culture experiments.

Previous studies have shown that overexpression of Smad7 reduces TGFβ-stimulated CCN2 gene expression, but has no effect on the basal expression of CCN2 [16]. However, ELISA analysis performed in our laboratory showed that CCD-1068SK fibroblasts secrete TGFβ in monocultures (results not shown), and it is therefore possible that Smad7 plays a role in negatively regulating autocrine TGFβ in these fibroblasts. Moreover, CCN2 has been shown to act as a co-mediator of TGFβ’s ability to promote type I collagen synthesis [18,25], suggesting that the decreased type I collagen gene expression observed in CCD-1068SK fibroblasts co-cultured with MDA-MB-231 tumour cells could occur as a result of the negative regulatory effect of increased Smad7 expression on CCN2 gene expression. Indeed, the siRNA experiments in CCD-1068SK fibroblasts showed that knockdown of CCN2 led to decreased levels of type I collagen, also confirming previous studies showing that changes in CCN2 expression can affect type I collagen gene expression in fibroblasts [19,21]. Smad7 overexpression has previously been shown to decrease COL1A1 mRNA levels in normal human fibroblasts [26], which supports our results obtained in fibroblasts directly co-cultured with tumour cells.

Transcription of Smad7 is known to be positively regulated by TGFβ signalling, leading to downstream inhibition of TGFβ/Smad signalling by Smad7 as part of a negative feedback loop [27-29]. Overexpression of Smad7 in tumour-associated fibroblasts may therefore result in their unresponsiveness to TGFβ signalling. Indeed, recent evidence suggests that fibroblasts unable to respond to TGFβ facilitate tumour growth [30]. By transplanting fibroblasts lacking the TGFβ receptor into mice together with mammary carcinoma cells, the aggressiveness and metastatic ability of the resulting tumours was shown to increase when compared to that observed in tumour cells transplanted together with normal fibroblasts. The altered fibroblasts produced TGFα and hepatocyte growth factor (HGF) which resulted in accelerated tumour cell growth. Since TGFβ also usually suppresses destructive immune and inflammatory responses [31,32], preventing the action of this tumour suppressor in breast cancer could result in tumour-promoting inflammatory conditions [23,33].

The upstream events leading to Smad7 overexpression in the herein described direct co-culture model of CCD-1068SK fibroblasts and MDA-MB-231 tumour cells has not yet been determined. Our results suggest that regulation occurs at the transcriptional level as Smad7 mRNA levels were found to be significantly increased. Previous studies investigating Smad7 regulation have mainly focussed on the effect of various cytokines on Smad7 expression. Those found to increase Smad7 levels include IFNγ via JAK/Stat signalling [34] and IL1β via either JNK or NFκB activation [35]. However, since Smad7 overexpression only occurred in fibroblasts directly co-cultured with tumour cells, this suggests that cell surface factors may be involved in regulation of Smad7. Further investigations would need to be performed to determine these factors.

Investigating the intracellular signalling events leading to CCN2 and type I collagen down-regulation, we found that tumour cell-mediated up-regulation of Smad7 negatively affected the MEK/ERK pathway. However, inhibition of this pathway had more dramatic effects on CCN2 expression while type I collagen was only slightly decreased. Previous studies have suggested that Ras/MEK/ERK signalling positively regulates CCN2 promoter activity and is required for basal CCN2 promoter activity [14,17,36,37]. However, the effect of MEK/ERK signalling on type I collagen gene expression is not clear. Some studies suggest that MEK/ERK activation negatively regulates type I collagen expression [38]. However, addition of IL-4 or IL-13 to dermal fibroblasts also increases type I collagen promoter activity in an ERK-dependent manner [39]. The effect of MEK/ERK signalling on type I collagen gene expression therefore appears to be dependent on interactions with other signalling pathways and on the cell context. Recent studies have shown that TGFβ-mediated up-regulation of both CCN2 and type I collagen in fibroblasts requires activation of Alk1(TGFBRI)/Smad1 and downstream ERK1/2 signalling [40] and that the association of CCN2 with β3 integrin is required for TGFβ-mediated Smad1 phosphorylation [25]. Silencing Smad1 gene expression resulted in a decrease in the expression of both TGFβ-stimulated CCN2 and type I collagen gene expression as well as basal type I collagen gene expression [40]. CCN2 has, in turn, been shown to activate ERK1/2 signalling by adhesion to the alpha1/beta6 integrin receptor or syndecan 4, a heparin sulphate proteoglycan [18,41]. The MEK/ERK signalling pathway therefore appears to play an important role in positively regulating CCN2 expression which, in turn, leads to further increased activation of MEK/ERK in a positive feedback loop. Deregulation of the MEK/ERK signalling pathway in fibroblasts close to or adjacent to tumour cells could therefore have important implications for ECM synthesis and homeostasis.

Previous studies have shown that levels of type I collagen gene expression were only decreased in later stages of breast tumour progression [42] and in melanoma tissue [43]. The negative regulation of tumour cells on CCN2 and type I collagen gene expression in fibroblasts may therefore be more likely to occur during the invasive stages of breast cancer, when tumour cells are in close contact with surrounding fibroblasts as a result of basement membrane degradation. Close association with invasive tumour cells could therefore cause the balance of ECM synthesis/degradation to be disturbed by decreasing the production of type I collagen and CCN2 in neighbouring fibroblasts and concurrently causing an increase in the expression of MMP1, a metalloproteinase that degrades type I collagen. Previous studies performed on highly invasive melanomas have shown that destabilization and degradation of the type I collagen matrix allows melanoma cells to evade the growth arrest and apoptosis that these cells would normally undergo in the presence of type I collagen matrix [43]. Inhibiting MMP expression in MDA-MB-231 cells has also been shown to inhibit the migration of these tumour cells through a bone marrow fibroblast monolayer [44]. The results obtained in these studies suggest that the decreased CCN2 and type I collagen matrix production and increased MMP expression observed in our model system of co-cultured CCD-1068SK fibroblasts could facilitate MDA-MB-231 tumour cell invasion through the ECM. However, further studies including primary human fibroblasts as well as breast tumour samples will need to be undertaken to support the observations described here.

## Conclusions

**Figure 5:**
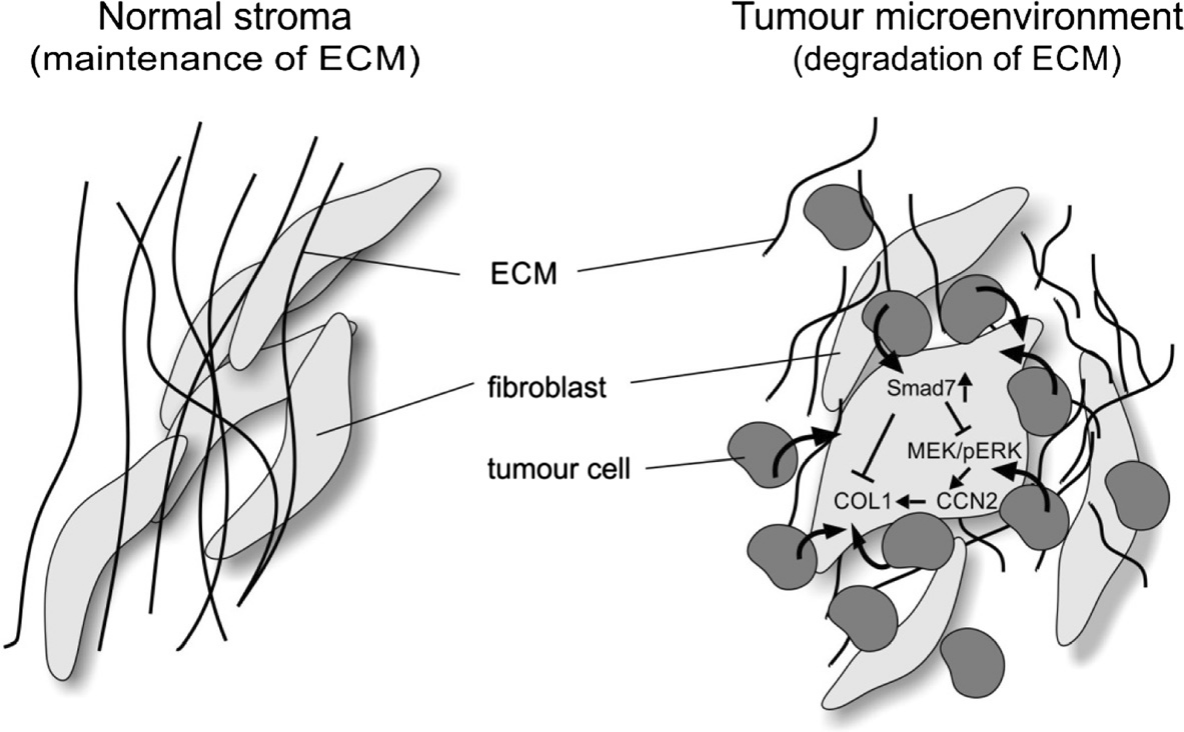
**Proposed model of breast tumour-mediated regulation of CCN2 and type I collagen in neighbouring fibroblasts in the tumour microenvironment.** While fibroblasts in the normal stroma maintain the structure and function of the ECM, direct contact of tumour-associated fibroblasts with breast tumour cells in the tumour microenvironment leads to degradation of the ECM and/or fibrosis. We hypothesise that this is caused by increased expression of Smad7 that downstream negatively affects the expression of CCN2 and type I collagen, partly through inactivation of the MEK/ERK signalling pathway (illustration by I. Jastram).

The co-culture model presented in this study revealed that tumour cells influenced ECM gene expression by direct cell-cell contact with fibroblasts. The observed effects were found to be mediated by increased levels of Smad7 that negatively influenced type I collagen and CCN2 expression, the latter occurring in a MEK/ERK dependent manner (Figure 5). To our knowledge, this is the first study showing a negative regulatory effect of Smad7 on CCN2 and type I collagen expression that is dependent on direct contact between fibroblasts and tumour cells. This type of close contact between tumour cells and fibroblasts is only possible in the later stages of breast cancer progression, when the basement membrane separating these two cell types has been degraded, and the resulting decrease in fibroblast-mediated production of the surrounding extracellular matrix could facilitate further tumour invasion and metastasis. Our results highlight the fact that invasive tumour cells may have effects on closely associated fibroblasts that would not occur under normal conditions and which could allow tumour cells to escape the inhibitory effects of the matrix, facilitating further tumour migration and invasion.

## Methods

### Cell culture

The cell lines CCD-1068SK (human breast fibroblasts), MDA-MB-231 (human breast tumour cells) and MCF12A (human non-tumorigenic epithelial cells) were purchased from ATCC and were grown in Dulbecco’s modified Eagle’s medium (DMEM, Invitrogen) supplemented with 10% (v/v) heat-inactivated fetal calf serum (FCS, Invitrogen), 100 μg/ml streptomycin and 100 U/ml penicillin (Invitrogen) in a humidified atmosphere (5% CO_2_) at 37°C.

### Direct co-culture

A total of 5×10^6^ CCD-1068SK fibroblasts were labelled with PKH67 green fluorescent dye (Sigma) in diluent C, according to the manufacturer’s instructions. After extensive washing, the fibroblasts were mixed with an equal number of MDA-MB-231 tumour cells, and 1.4×10^6^ cells were seeded into 150 cm dishes. In parallel, 1.4×10^6^ CCD-1068SK fibroblasts were seeded into separate 150 cm dishes and served as a control. Cells were allowed to settle in complete medium for at least 12 hours before being washed twice with 1×PBS and incubated in serum-free medium for a further 48 hours.

### Indirect co-culture

CCD-1068SK at a density of 2×10^5^ cells/well were seeded into 6-well plates while an equal number of MDA-MB-231 or CCD-1068SK cells were seeded on transwell inserts (NUNC, membrane pore size 0.2 μm) in separate 6-well plates. Cells were allowed to settle in complete medium for at least 12 hours before inserts were transferred into the 6-well plates containing the fibroblasts. Medium was removed, cells were washed twice with 1×PBS and incubated in serum-free DMEM for a further 48 hours.

### Fluorescence-activated cell sorting (FACS)

Directly as well as indirectly co-cultured cells were lifted with 0.05% trypsin/5mM EDTA (pH 8), washed with complete medium and prepared for FACS in DMEM containing 2% FCS. CCD-1068SK fibroblasts were sorted based on green fluorescence using the BD FACS VANTAGE, collected in DMEM containing 2% FCS and used for further RNA and protein analysis.

### Oligo GEArray® human extracellular and adhesion molecules microarray analysis

RNA was extracted from CCD-1068SK fibroblast using the RNeasy® MinElute™ Cleanup Kit (Qiagen), according to the manufacturer’s instructions. The TrueLabeling-AMP™ 2.0 kit (SABiosciences) was used to synthesize cDNA from 3 μg of each RNA sample. The amplified cDNA then formed the template for further cRNA synthesis, also using the TrueLabeling-AMP™ 2.0 kit. The cRNA was purified using the ArrayGrade™ cRNA Cleanup Kit (SABiosciences) and hybridized against Oligo GEArrays® nylon membranes overnight at 60°C with continuous rotation. Binding of biotinylated cRNA probes was detected using alkaline phosphatase-conjugated streptavidin together with the Chemiluminescent Detection Kit (SABiosciences). Array images were visualized using the Syngene G:Box Chemi system. The images were uploaded onto the web-based GEArray Expression Analysis Suite for further analysis. The microarrays were done in duplicate, background was normalized against two empty spots on each array and gene expression was normalized against ribosomal protein S27a (RPS27A) and β-actin (ACTB) gene expression.

### Quantitative real-time PCR

Total RNA was isolated from CCD-1068SK fibroblasts using Qiazol reagent (Qiagen) according to the manufacturer’s protocol and reverse transcribed using the ImProm-II™ Reverse Transcription System (Promega). cDNA generated from 1 μg of total RNA was used for quantitative PCR with the KAPA SYBR® FAST qPCR Kit (KAPA Biosystems) and the relevant primer sets (Table 2) on a LightCycler® 480II System (Roche). To determine relative gene expression, results were analysed using the 2^-ΔΔC^T method [45] and normalised to GAPDH expression.

**Table 2:** Primers used for quantitative real-time PCR

**Gene name**	**Primers**	**Annealing temp.**	**Product size**	**Ref.**
GAPDH	F: GGCTCTCCAGAACATCATCC	60°C	192 bp	This study
	R: GCCTGCTTCACCACCTTC			
COL1A1	F: CAGCCGCTTCACCTACAGC	60°C	83 bp	[46]
	R: TTTTGTATTCAATCAGTGTCTTGCC			
COL1A2	F: GATTGAGACCCTTCTTACTCCTGAA	60°C	78 bp	[47]
	R: GGGTGGCTGAGTCTCAAGTCA			
CCN2	F: GTTTGGCCCAGACCCAACT	60°C	650 bp	This study
	R: GTGCAGCCAGAAAGCTCAAA			
MMP1	F: ATCCACTCCCCATTTCACAA	60°C	867 bp	This study
	R: TCCTGCAGTTGAACCAGCTA			
Smad7	F: CCAGATAATTCGTTCCCCCTGT	60°C	137 bp	[48]
	R: CCTTAGCCGACTCTGCGAACTA			

### Western blot analysis

Cells were lysed in 1×RIPA buffer (Invitrogen) containing 1×protease inhibitor (Roche) and 1×phosphatase inhibitor (Roche), and quantitated using the BCA™ Protein assay kit (Pierce). Approximately 20 to 30 μg of protein was heat-denatured at 95°C, separated via SDS-polyacrylamide gel electrophoresis and transferred to a nitrocellulose membrane. Membranes were blocked in 5% milk in TBS-Tween (TBST) for 1 hour and probed with the following primary antibodies at 4°C overnight: CTGF/CCN2 (L-20) (Santa Cruz Biotechnology), Smad7 (G-23) (Santa Cruz Biotechnology), type I collagen (Southern Biotech), pERK1,2 (T202/Y204) (Cell Signalling), Erk2 (Santa Cruz Biotechnology), and β-tubulin (Santa Cruz Biotechnology). After washing with TBST, membranes were incubated with the appropriate secondary antibody for 1 hour at room temperature. Protein levels were visualized by chemiluminscence using the LumiGlo® Reserve Substrate (KPL) and the VisionWorks LS Biospectrum™ 500 Imaging System (UVP).

### Transient transfections

CCD-1068SK fibroblasts were plated at a density of 2×10^5^ cells per well in 6-well plates and allowed to settle overnight to reach a final confluence of ca. 50%. For gene-knockdown experiments, Transfectin lipid reagent (Bio-Rad) was added in a 2:1 ratio to 20–80 μM CCN2 siRNA or 80 μM Smad7 siRNA (Dharmacon), respectively, in serum-free DMEM and incubated at room temperature for 20 min before being added drop-wise to the cells. Cells were incubated overnight, medium was changed to serum-free DMEM and cells were incubated for a further 24 hours before continuing with RNA and protein extractions as described above. CCD-1068SK fibroblasts transfected with an equal amount of scrambled control siRNA-A (Santa Cruz Biotechnology) were used as a negative control.

To transiently overexpress Smad7, 1 μg of the plasmid pORF9-hSmad7 (InvivoGen) in 150 mM NaCl was added to 2 μl JetPEI® reagent (Polyplus) in 150 mM NaCl and incubated at room temperature for 20 min. A total volume of 200 μl transfection mixture was then added drop-wise to the cells. 8 h and 48 h post transfection, RNA and protein were extracted from the cells and used for further analysis as described above.

### Analysing the incorporation of [^3^H]-proline into secreted α1(I) and α2(I) procollagen

CCD-1068SK fibroblasts at a density of 2×10^5^ cells were mixed with an equal number of MCF12A or MDA-MB-231 cells, seeded into 6-well plates and allowed to settle overnight. Cells were then washed twice with 1×PBS, after which 2 ml serum-free DMEM with 20 μCi/ml [^3^H]-proline (American Radiolabeled Chemicals Inc), 50 mg/ml ascorbic acid and 50 mg/ml β-aminopropionitrile was added to each well and incubated for 20 hours.

Medium was removed from cells, transferred to 2ml microfuge tube and acetic acid was added to a final concentration of 0.5 M. Medium proteins were digested with 100 μg/ml pepsin for 4 h at 20°C, with rotation. Digested medium was transferred to dialysis tubing and dialyzed overnight against 50 mM Tris, pH 7.5, with one buffer change after 2 hours. Medium was transferred back into microfuge tubes and precipitated with TCA overnight at 4°C. The samples were centrifuged at 11 000 rpm for 15 min, washed twice with acetone, air-dried and dissolved in 40 μl of SDS-Page loading buffer. An equal volume of each sample was heat-denatured at 95°C for 5 minutes and run on an 8% SDS-PAGE gel (with 4% stacking gel) for 80 minutes at 180 V. The gel was soaked in 1M sodium salicylate for 1 hour, washed in distilled water for another hour and placed on 3 mm Whatman paper, covered with saran wrap and vacuum dried at 70°C for 2 hours. The dried gel was placed in a cassette and exposed to film for 7 days at -80°C, after which it was developed and fixed.

### Statistical analysis

All experiments were performed in triplicate and repeated at least twice. Standard deviations (SD) were calculated from triplicate samples by means of the two-tailed Student’s t-test and a p-value ≤ 0.05 was regarded as statistically significant.

### Ethical approval

No animal or human work has been performed in this study; therefore no ethics committee approval was required.

## Abbreviations

ECM: Extracellular matrix; CTGF: Connective tissue growth factor; TGFβ: Transforming growth factor beta.

## Competing interests

The authors declare that they have no competing interests.

## Authors’ contributions

BAR and GS participated in the design of the study, carried out the molecular and biochemical experiments and drafted the manuscript. MIP conceived the study. VDL and MIP participated in the design of the study and coordination and helped to draft the manuscript. All authors read and approved the final manuscript.

## Supplementary Material

Additional file 1: Table S1 Genes included on the Extracellular Matrix and Adhesion Molecules OligoGEArray. Click here for file
