# Supplementary material for: Tumour cells down-regulate CCN2 gene expression in co-cultured fibroblasts in a Smad7- and ERK-dependent manner
Source: Cell Commun Signal. 2013 Oct 3;11:75. doi: 10.1186/1478-811X-11-75 (PMC3850759; doi:10.1186/1478-811X-11-75)
Supplement: Additional file 1: Table S1 — Genes included on the Extracellular Matrix and Adhesion Molecules OligoGEArray. [file 1478-811X-11-75-S1.pdf]

Supplementary Table 1. Genes included on the Extracellular Matrix and Adhesion Molecules OligoGEarray.

Abbreviations: HAS, hyaluronan synthase; ICAM, intercellular adhesion molecule; NCAM, neural cell adhesion molecule; PECAM, platelet endothelial cell adhesion; VECAM, vascular endothelial cell adhesion molecule; SELE, E-selectin; SELL, L-selectin; SELP, P-selectin; ITGA, integrin α; ITGB, integrin β; MMP, matrix metalloproteinase; SGCE, episilon-sarcoglycan; SPG7, paraplegin; COL, collagen; CTNN, catenin; ADAMTS, A disintegrin and metalloproteinase with thrombospondin motifs; SPP, stromal processing peptidase; THBS, thrombospondin; CNTN, contactin; VCAN, versican; FN, fibronectin; KAL1, anosmin-1; LAMA, laminin α; LAMB, laminin β; CLEC, Tetranectin; VTN, vitronectin; TIMP, tissue inhibitors of metalloproteinases; ECM, extracellular matrix protein.

**Transmembrane Molecules**	CD44, CDH1, SGCE<br>HAS1,<br>ICAM1, NCAM1, PECAM1, VCAM1,<br>SELE, SELL, SELP<br>ITGA1, ITGA2, ITGA3, ITGA4, ITGA5, ITGA6, ITGA7, ITGA8, ITGAL, ITGAM, ITGAV, ITGB1, ITGB2, ITGB3, ITGB4, ITGB5,<br>MMP14, MMP15, MMP16, SPG7
**Cell-Cell Adhesion**	CD44, CDH1,<br>COL11A1, COL14A1, COL6A2,<br>CTNND1,<br>ICAM1, VCAM1<br>ITGA8
**Cell-Matrix Adhesion**	ADAMTS13,<br>CD44, SGCE<br>ITGA1, ITGA2, ITGA3, ITGA4, ITGA5, ITGA6, ITGA7, ITGA8, ITGAL, ITGAM, ITGAV, ITGB1, ITGB2, ITGB3, ITGB4, ITGB5,<br>SPP1,<br>THBS3.
**Other Adhesion Molecules**	CNTN1, VCAN, CLEC3B<br>CCN2, THBS1, THBS2, TNC<br>CTNNA1, CTNNB1, CTNND2,
**Collagens & ECM Structural Constituents**	COL11A1, COL12A1, COL14A1, COL15A1, COL16A1, COL1A1, COL4A2, COL5A1, COL6A1, COL6A2, COL7A1, COL8A1,<br>FN1,<br>KAL1.
**ECM Proteases**	ADAMTS1, ADAMTS13, ADAMTS8,<br>MMP1, MMP10, MMP11, MMP12, MMP13, MMP14, MMP15, MMP16, MMP2, MMP3, MMP7, MMP8, MMP9,<br>SPG7,<br>TIMP1
**ECM Protease Inhibitors**	COL7A1,<br>KAL1,<br>THBS1,<br>TIMP1, TIMP2, TIMP3
**Basement Membrane Constituents**	COL4A2, COL7A1,<br>LAMA1, LAMA2, LAMA3, LAMB1, LAMB3, LAMC1,<br>SPARC.
**Other ECM Molecules**	VCAN, CLEC3B<br>CCN2, THBS2, THBS3, TNC<br>ECM1, VTN<br>HAS1, SPP1,<br>TGFBI
